# Indium Antimonide Nanowires: Synthesis and Properties

**DOI:** 10.1186/s11671-016-1370-4

**Published:** 2016-03-24

**Authors:** Muhammad Shafa, Sadaf Akbar, Lei Gao, Muhammad Fakhar-e-Alam, Zhiming M. Wang

**Affiliations:** Institute of Fundamental and Frontier Sciences, University of Electronic Science and Technology of China, Chengdu, 610054 People’s Republic of China; Zernike Institute for Advanced Materials, University of Groningen, 9747AG Groningen, The Netherlands; State Key Laboratory of Electronic Thin Film and Integrated Devices, School of Microelectronics and Solid-State Electronics, University of Electronic Science and Technology of China, Chengdu, 610054 China

**Keywords:** Indium antimonide nanowires (InSb NWs), Growth parameters, Applications

## Abstract

This article summarizes some of the critical features of pure indium antimonide nanowires (InSb NWs) growth and their potential applications in the industry. In the first section, historical studies on the growth of InSb NWs have been presented, while in the second part, a comprehensive overview of the various synthesis techniques is demonstrated briefly. The major emphasis of current review is vapor phase deposition of NWs by manifold techniques. In addition, author review various protocols and methodologies employed to generate NWs from diverse material systems via self-organized fabrication procedures comprising chemical vapor deposition, annealing in reactive atmosphere, evaporation of InSb, molecular/ chemical beam epitaxy, solution-based techniques, and top-down fabrication method. The benefits and ill effects of the gold and self-catalyzed materials for the growth of NWs are explained at length. Afterward, in the next part, four thermodynamic characteristics of NW growth criterion concerning the expansion of NWs, growth velocity, Gibbs–Thomson effect, and growth model were expounded and discussed concisely. Recent progress in device fabrications is explained in the third part, in which the electrical and optical properties of InSb NWs were reviewed by considering the effects of conductivity which are diameter dependent and the applications of NWs in the fabrications of field-effect transistors, quantum devices, thermoelectrics, and detectors.

## Review

### Introduction

The fifteenth anniversary of indium antimonide nanowires (InSb NWs) synthesis was recently monumentalized a good opportunity to review, try to discuss and compile few of its imperative aspects of synthesis, of the growth thermodynamics, of optical properties, and electrical characterizations. The proclamation of a fifteenth anniversary invoked from the synthesis of S.V. Z. Zotov et al. [1] indium antimonide structure which, as far as we know, shows the earliest communication on InSb NWs synthesis, in which the authors declare effective growth of NWs having 50 Å diameter and 1 mm long. This growth technique has been deduced from the vapor–liquid–solid (VLS) mode which was introduced in the 1960s–1970s for large whisker growth [2, 3]. During that period, the terminology of whisker was commonly used for fiber-like grown structures corresponding to silicon crystals in the dimension of micrometer (see, e.g., the magnificently long wires shown in [4]). Furthermore, nomenclatures of nanorods have also been utilized instead of terms whisker [5]. Classical name whisker has not been exploited throughout this communication, even though previous studies of these nanostructures were reviewed. Alternatively, the term InSb NWs will be used corresponding to the diameter of wire that was even smaller than a tenth of nanometers. During a generalized way of descriptions, we will use the renowned term NWs which is not limited to a specific size. We will confine our self to the said nomenclature, although not with extreme strictness.

**Fig. 1:**
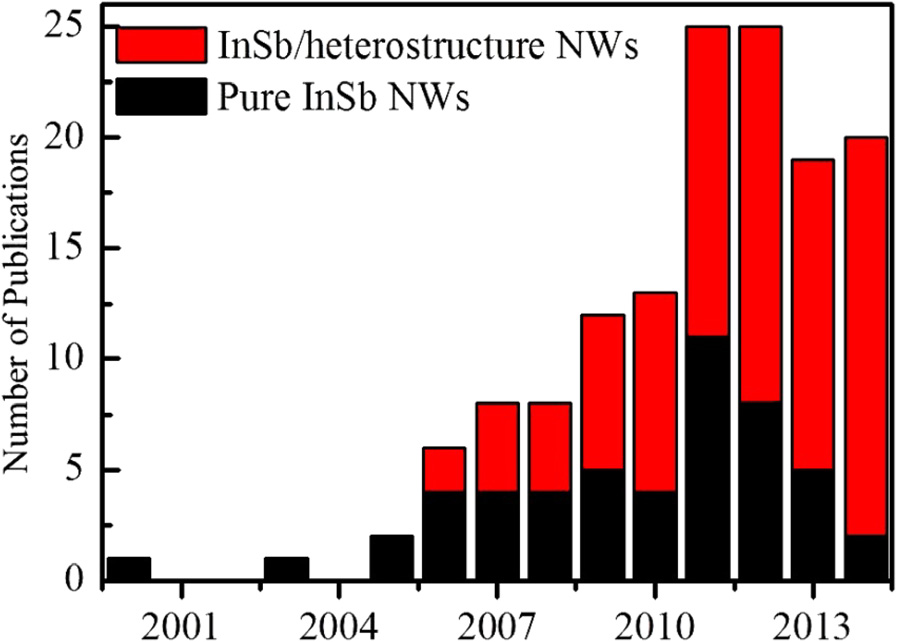
Histogram of the pure InSb NW publications as a function of the years in which they were published. Source: ISI Web of Knowledge ^(SM)^; search date 12th December, 2015

Looking back in the history, 1960s, four decades after the studies of Treuting and Arnold were published [6], they did an investigation on NWs and boost up thenceforth a mechanism significantly developed by the pioneering work of S.V.Z.Zotov et al. [1]. In this paper, they claimed evaporation of InSb powder under high pressure for growth of crystalline NWs helps to establish a new horizon in the optoelectronic industry and a most economical way to synthesize single crystalline InSb NWs. As demonstrated in Fig. 1, InSb NW research fundamentally began with the publication of S.V.Z.Zotov et al. [1], flourished for about 15 years. However, during that period, a lot of fundamental aspects of VLS InSb NWs growth discovered [7]. The second period in InSb NW synthesis begins in the mid 2005s, when boost in optoelectronic devices launched a modern interest in InSb NWs analysis. Xueru Zhang et al. [8] used an electrodeposition method for growth of wires having truly nonaoscopic dimension which is a new technique for InSb NW fabrication. Outnumbered one dimensional publications on the growth of InSb NW research illustrated in Fig. 1 and studied on InSb NWs experienced an incredible increase in magnitude and endorsement until now. With low band gap [9] and high mobility [10], it becomes a significant choice as the electronic material for infrared device fabrication, it is obvious due to its wide applications at forthcoming optoelectronic devices. A major focus of InSb NW research was its optoelectrical properties; as a result, NW synthesis was a major issue to be addressed on prior basis. Which is also being the objective of this paper, this review provides a concise compendium with detail on different techniques of InSb NW fabrication as well as of their optoelectrical applications, commencing with the VLS growth techniques as it is at the heart of NW investigation. Following thereupon, a concise abstract of numerous InSb NW fabrication techniques demonstrated previously is described. With the adaptation of these NWs for the optoelectronic industry, the catalyst materials used as seed for their growth are critical; a recent situation of research concerning the gold [11–16] as catalyst, silicon oxide [17–20], and self-catalyzed [21–25] materials is discussed. Thereafter, for more detailed experimental parts, we discussed four major thermodynamic effects for VLS NW synthesis, which are, first, theoretical modeling for the expansion of the grown NWs, because it rationally explains the interplay between droplets and NWs. Second, thermodynamics of NW growth criterion concerning the expansion of NWs, growth velocity, Gibbs–Thomson effect, and growth model is described in detail. Third section, the growth model of NWs is discussed in detail. Finally, we will focus on the electrical and optical properties, discuss the effects essential for the conductivity of InSb NWs which are diameter dependent, and also discuss applications of NWs for the synthesis of field-effect transistors, quantum devices, thermoelectrics, and detectors. On the other hand, when trying to grow InSb non-planar nanostructures, there is the way to obtain also some 2D materials in the form of InSb nanosheets on top of the InAs stem [26, 27]. In some cases, the difference between growing a simple nanowire or one of these 2D-type stand up nanostructures lays in the initial formation of a twin defect due to some instability during the growth [26, 28], as the present review pretends to give a detailed overview of the whole InSb nanowire-related literature.

### Methods and Materials for InSb NW Growth

#### Vapor–Liquid–Solid Mechanism

**Fig. 2:**
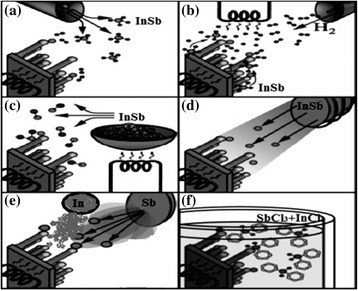
Experimental setups for growth of InSb NWs. **a** Chemical vapor deposition, **b** annealing in reactive atmosphere, **c** precursor (In/Sb) evaporation, **d** molecular beam epitaxy, **e** chemical beam epitaxy, and **f** hydrothermal (solution-based) growth

As mentioned, in the mid-1960, Wagner and Ellis [2] are the pioneers who presented vapor–liquid–solid (VLS) growth mechanism, which is the key technique recently adopted for InSb NW growth as well. VLS mechanism proposed by them are rooted on the two major conclusions: metallic impurity know as catalyst is obligatory for the synthesis of InSb NWs in experiments, and these catalyst particles must reside in the tip of the NWs during growth. From these results, Wagner and Ellis inferred that metallic seeds residing at the tip of the NWs play the role of sink for precursors during the growth or it might be a chemical process that is involved in growth [29]. When a thin film of gold, for example, is grown on a substrate by any means and this substrate annealed rapidly at the temperature of 400 °C [30], as result, an island of the gold droplet forms on the surface of the substrate surface. When this substrate containing the gold island is exposed to a gaseous indium and antimonide precursors, such as trimethylindium (TMIn) and trimethylantimony (TMSb) [30], precursor particles will crack on the upper surface of the gold droplets, as a result InSb is integrated into the gold droplet. Due to the gaseous phase of precursor supply, the gold droplet becomes supersaturated with indium antimonide until it solidifies at the indium antimonide/droplet interface. Similarly, we can use other precursors like tertiarybutylarsine (TBAs) and tertiarybutylphosphine (TBP) [23] for heterostructured growth. As it is well known, most of the approaches used for growing InSb nanowires use InAs or other semiconductor nanowires as stems. In this way, a recent approach study was published on the strain relaxation mechanism on such heterointerfaces [31]. Synthesis of the NWs take place if this method is continued depending upon the growth time while the gold droplet arises atop [2] as shown in Fig. 2. During growth of NWs by VLS growth mechanism, precursors in the form of the vapor phase deposited through particles generally known as catalyst and finally end up as a solid. Apart from the VLS growth mechanism, many other techniques for the synthesis of NWs were also demonstrated and named accordingly. In the context of it, vapor–solid–solid (VSS) is another successful technique for growth of NWs, which is initiated with solid catalyst particle instead of a liquid droplet. It is clear that the growth temperature as well as type of catalyst materials play a major role on whether NW growth proceeds via a VLS or VSS route. Furthermore, as regards the growth from both prior mechanisms, it is hard to evaluate as well as difficult to describe which of the two growth mechanisms dominate. Hence, the shape of catalyst particles can give information about the mechanism of growth whether it is VLS or VSS as well as the dynamics of NWs. It is commonly observed that during VLS NW growth, the size of the droplet radius is directly proportional to the radius *r* of grown NWs. In equilibrium, it can be deduced that, $R=r\sqrt{1/\left(1-{\left({\delta}_{1S}/{\delta}_1\right)}^2\right)}$ [32] where *δ*_1*S*_ and *δ*_1_ are interface tension between solid/liquid and surface tension of the catalyst, respectively. But the significant characteristics of the VLS techniques is that it can grow a wide range of wires from few nanometer to several hundreds of micrometers thick [2] in diameter.

#### Nanowire Growth Techniques

Before exploring NW growth in depth, a concise summary of the various growth techniques is described in the following section.

##### Chemical Vapor Deposition

Like other synthesis techniques, the word chemical vapor deposition (CVD) comprises of three words, chemical, vapor, and deposition; it means that first precursors are converted into vapor phase, of course at high temperature, and then it is deposited under the flow of carrier gas. In CVD, during growth of InSb NWs, gaseous indium antimonide precursors, such as trimethylindium (TMIn) and trimethylantimony (TMSb) [15, 24], play the role of source materials. It is transported with the help of some carrier gas to the substrate surface at which these source materials react chemically, and it distributes itself into their ingredients as demonstrated in Fig. 2a. Basically, highly crystalline thin films are grown form CVD. To enhance the growth process, gold particles were used as catalyst; however, it may cause anisotropic growth of InSb NWs. CVD permits epitaxial synthesis of InSb NWs, where the growth velocity varies with molar fraction of III/V ratios from 5 to 12 [20] depending on what type of III/V precursors and growth temperature utilized. Furthermore, CVD offers a broad possibility of modifying the properties of the NWs in a controlled manner [32]. A large variety of CVD methods exist which can be categorized by different parameters such as the treatment of the precursor as well as base and operating pressure or since it is a well-known fact that precursors are oxidize quickly which is major issue needed to be resolved first before growth of crystalline InSb NWs starts. Complete removal of oxygen is impossible, but it can be reduced. Specially, it is worth noticing that oxygen-sensitive catalyst materials are advantageous to use for the growth of NWs; due to this significant property, growth can be carried out without exposing growth chamber to ambient during synthesis [33]. To reduce the oxidation significantly during growth, we have to have ultra-high or very high vacuum which facilitates the growth process at elevated temperatures as well as lessens the role of unwanted contaminations [34].

The amount of precursors transported to the growth surface usually depends upon the cracking probability at the surface of catalyst and source temperature. Synthesis of InSb NWs, must not be but it can be executed at significantly low pressures where highly pure hydrogen gas is used as carrier gas and antioxidant [35, 36] with molar fraction of 1.1 × 10^−5^ for TMIn and 3.4 × 10^−3^ for TMSb [37]. Growth can be modified by controlling the precursor before depositing on the substrate surface just by regulation of growth temperature. The situations where droplets must be highly supersaturated or the thermal load is vital, NW synthesis can be enhanced by using metal-organic CVD (MOCVD) [17, 23, 30, 38, 39]. Bottom-up synthesis mechanism is another superiority of CVD in which its variability can be enhanced concerning the intended NW dimensions whereas NW diameter vary from below 10 nm [40–42] up to tens of micrometers in length. In the CVD chamber, the surface diffusions play minor role during the growth of NWs so length of the NWs can be easily tunable simply by increasing or decreasing growth time. Thus, to summarize, CVD is a very effective technique which is used for the synthesis of NWs with tuned diameter and length configurations in a wide range [22] as well as properties of these grown NWs can also be controlled with the help of this technique. A phenomenon of controlled doping can also be carried out with the help of CVD; for intentionally introducing additional doping materials as well as by switching doping precursors, we can generate doping profiles in any direction. A major drawback of CVD for the growth of InSb NWs is the growth direction which cannot be tuned, as several studies have shown that InSb NWs normally grow in a <111>B direction [43], depending upon the available direction of those on substrate [37]. To overcome this hurdle, template such as anodic aluminum oxide (AAO) [44] is used for the growth of InSb NWs where the pore of the templates is filled with catalyst materials to stimulate the NW growth only inside the pores. In this way, NW growth is controlled especially epitaxial growth oriented at (200) which is impossible for free standing growth to be achieved by using this template method. After the growth is done, the template in which these NWs are grown can be washed out with the solution of sodium hydroxide; as a result, standing NWs obtained can be shifted for further characterizations [44].

##### Annealing in Reactive Atmosphere

Before 2003, a technique to grow InSb whisker microcrystal free crystallization from a gaseous phase [45] with the initial iodine pressure of 375 Torr heated source up to 760 °C is already pioneered; at growth temperature, the carrier gases can react in the growth chamber with the precursors that can be doped with several impurities simultaneously (Sn, Al, and Cr) during their growth [46]. This kind of addition of impurity as metal droplet acts as seed to start the synthesis of NWs also observed in the conventional CVD. Being cheap and technically simple are major superiorities of this mechanism, which is surly the logic why it was adopted for InSb microcrystal growth elongated along the (111), (110), and (211) with the dimension of (10 × 0.06 × 0.02) mm [45]. In some sense, this technique can be recognized as the ancestor of NW synthesis by traditional CVD. Nowadays, a modification in this technique is only hot-filament CVD which is schematically demonstrated in Fig. 2b [40].

##### Evaporation of Indium Antimonide

To vapor the indium antimonide powder is the cost-effective technique for the growth of InSb NWs on a massive scale whose schematic diagrams are shown in Fig. 2c. Simplistic way to vaporize precursors is a use of multiple-zone tube furnace supplied with inert gas (helium or argon), and precursors in the form of InSb powder are the primary requirements for the growth of NWs. Temperature gradient is a critical factor which generally varies from growth temperature of about 1100 to 400 °C along the furnace tube for vaporization of precursor. InSb powder is placed at high source temperature of 550 °C and evaporated under the flow of gas stream towards seeded substrates placed at significantly low temperature of 470 °C which is called growth temperature in downstream, where precursors undergo a nucleation reaction; as a result, NWs are synthesized [47, 48].

In principle, growth with and without a metal catalyst are the two permissible growth methods. It is generally observed that NW growth is rapid in the presence of a catalyst [35] in accordance with the fact of vapor–liquid–solid synthesis mechanism where the radius of NWs is measured by the size of the catalyst particle; although compared to conventional CVD growth, the interplay between the NWs and metallic catalysts seems to be more complicated. Due to the phenomenon of nucleation reaction, diametric ratio of NW crystalline core and the shell residing on the top remains constant [10, 49].

Catalyst-free growth, second phenomena for NW growth via tip-led process of individual NW, is necessary for avoiding contaminations in metal-organic chemical vapor depositions [24, 50, 51], where it was observed that NWs can be grown by using antimony cluster as catalyst on their tip. Remarkable about this layer-assisted growth is that the yield of the final amount of InSb NW length as well as diameter is sufficiently high [50] compared to metal-assisted growth [36]. For several micrometer-long crystalline InSb NWs with diameters varying from about 300 to 500 nm and 4–5 μm in length, the growth process should be carried out for a longer time which may also increase the diameter of the NWs, enclosed with amorphous coating of precursors up to few 10 nm [50].

##### Molecular Beam Epitaxy

In this technique, a highly pure solid indium and antimony sources are heated until it start to evaporate. Figure 2d schematically represents a molecular beam epitaxy (MBE) configuration. During growth, controlled gaseous beams of indium and antimony atoms are targeted at the substrate, on which the precursor’s atoms crystallize as well as adsorb. Base pressure should be kept at ultra-high vacuum during the growth, to diminish the effect of contamination, while reflection high-energy electron diffraction monitor the growth of thin films [52] or low-energy electron-beam diffraction to investigate the surface structure. Analogous to CVD, this technique was also designed for layer by layer epitaxial growth only. With the passage of time, metal contamination was introduced on substrate to cause NW growth similar with CVD, due to nucleation of precursor gases at the surface of the liquid metal–alloy. Hence, in the recent ages, this technique becomes very conventional and can be used as a traditional seed particle to stimulate the growth. In the chamber, two fluxes have been used for the growth of InSb NWs. First, the indium is vaporized from the solid source; and the other one is the antimony in vapor state which comes out from solid sources in growth chamber equipped with ultra-high vacuum. The NWs fabricated by MBE—generally grown on InP(111)B substrates—are epitaxially oriented at (111) [52]. MBE provides magnificent flexibility to limit incoming flux, such that pure InSb NWs or heterostructures [52, 53] can be synthesized just by changing the flux of sources. In fact, MBE could be used to grow high-quality ultrathin InAs NWs [54]. Very recently, thin InSb NWs with diameter down to 30 nm in a heterostructure with an InAs NWs are realized by MBE [27], which seems to be a consequence of the Gibbs–Thomson effect, and the fact that only small supersaturation are attainable by MBE. Major drawback of MBE growth is the very slow growth rate of NWs, that is, we can grow only few nanometers of NWs in length per minute [52].

##### Chemical Beam Epitaxy

In this synthesis mechanism, a highly pure solid antimony source and gaseous Indium sources are used as shown in the Fig. 2e. The InSb NWs grown by chemical beam differ in many aspects from the NWs grown by MBE, as CBE have digital mass flow controller with a high growth rate, while in contrast to MOVPE, the temperature window is significantly broad [55] and there is no need for carrier gas [56]. In CBE, metal-organic precursors are directly induced into a chamber without use of carrier gas. TMIn and TESb are used as precursors, where TESb is pre-cracked at 625 °C to get homogenous compositions [55]. TMIn, in contrast, is introduced into the growth chamber through an injector held at about 70 °C. The decomposition of TMIn starts at temperatures around 300 °C [57]. Following the VLS growth technique, just after supersaturation of the catalyst with InSb atoms, NWs start to grow and this growth proceeds as long as liquid phase of catalyst remains. NWs grown by CBE method have a lot of advantages: first, there is a high growth rate, and due to low pressure a ballistic mass is transported to substrate. Second, the ingredients of the grown NWs can be adjusted with the help of digital mass controller [58]. Ex situ high-resolution transmission electron microscopy (HRTEM) used to investigate the defects in NWs that are grown by CBE [58].

##### Solution-Based Techniques

Synthesis of NWs can also be done within liquid media for which vapor phase is not necessary. The major drawbacks with the abovementioned techniques are of a fundamental difficulty in achieving a controllable stoichiometric growth of InSb NWs which are a narrow range of temperature processing, formation of the thin In_2_O_3_ layer on the surface of NWs, and differences in vapor pressure of In and Sb [59, 60]. To avoid all these complications, solution methods for synthesis of InSb NWs with controlled stoichiometry and high crystallinity are desirable. As one of the promising alternative strategies to avoid these problems and synthesize high crystalline InSb NWs, the electrochemical deposition inside nanoporous anodic aluminum oxide (AAO) templates has been considered [8, 40, 61–63]. These solution-based synthesis methods are preferred for high-yield InSb NWs. Such kind of methods utilizes supercritical organic fluids which are highly pressurized, enriched with a liquid indium and antimony as precursors, such as antimony trichloride and indium(III) chloride, and nanoparticles of metal catalyst [44, 64], as shown in Fig. 2f. In this phase diagram, at temperatures over the metal–InSb eutectic, the precursors decompose and form composite with gold that act as a working electrode. By comparing this method with VLS growth technique, the composite seed catalysts in this supercritical–fluid–liquid–solid (SFLS) mechanism originate the growth of InSb NWs once the composite droplet acquired supersaturated phase with indium antimonide [39]. Using this approach, one can synthesized crystalline NWs with diameters up to 50 nm and several micrometers long [8]. Identical to the VSS synthesis technique, growth of InSb NWs via a metallic seed particle has also been indicated for the liquid-based technique in which micrometer-long NWs were grown at a temperature of merely 550 °C under pressure of 15 kbar using gold as catalysts [1]. Solution–liquid–solid (SLS) method is another high-yielding NW production method. By using this method, under atmospheric pressure, an organic solvent is used for the synthesis of micrometer-long crystalline NWs with 50 nm in diameter, which has been demonstrated [61]. This cost-effective techniques for NW production, as it can be carried out with a sufficiently less expensive apparatus.

##### Top-Down Fabrication Methods

In the preceding section, a lot of discussions have been made for bottom-up growth mechanism while several attractive top-down synthesis methods also exist for the growth of single crystalline InSb NWs. Due to the diversity in the growth techniques, one should easily differentiate between the fabrications of horizontal NWs, that is, NWs lying in the substrate plane with the growth of vertical NWs, that is, NWs oriented perpendicular to the substrate. During the top-down fabrication method, InSb NWs are mostly fabricated by focused ion beam irradiation of InSb wafer [65, 66] or by lithography and then etching steps, with the help of reactive ion etching after electron-beam lithography [67]. This phenomenon of lithography is simply described here because the detail of it is far from the contents of this article. The interested reader is referred to the well-renowned articles of J. H. Wu and R. S. Goldman [65] and the references therein. In most cases, on irradiation of InSb surface, cone-shaped nanorods evolved are capped with In islands. During irradiation, with the diameter of nanorods increasing in ion energy, the cone-shaped nanorods transformed to capless nanorods with a truncated cone shape. These results suggest a growth mechanism in which both the nanorod cap and body are supplied by redeposition of atoms sputtered from the InSb substrate using 3–30 KeV ion beam energy during irradiation [65, 66]. Lithography step can also be defined as the diameter of NWs/nanorods which varies from 200 to 400 nm followed by reactive ion etching. Numerous nanofabrication techniques, such as nanosphere lithography [68], electron-beam lithography [37], colloidal dispersion [69], are also used for the fabrication of NWs.

##### Summary

**Fig. 3:**
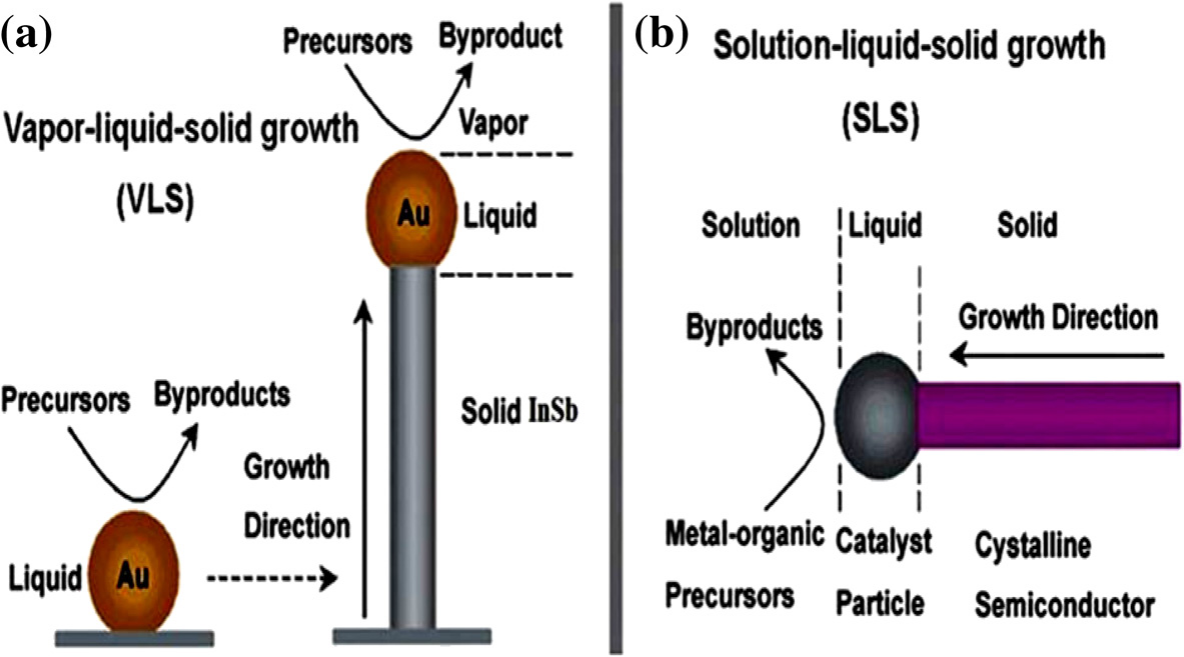
Two typical growth mechanisms of semiconductor NWs: **a** vapor–liquid–solid growth; **b** solution–liquid–solid growth. See ref. [179]

The various growth techniques discussed in the previous sections can be distinguished on different aspects, and the question on which method is used to adopt the synthesis totally depends on the applications, that is, which device one has to fabricate these NWs. More concisely, it is note worthy to fabricate the device on the same substrate on which NWs are grown, that is, direct fabrication of the nanodevice. On the other hand, it is a more challenging job to fabricate a device and then shift to another stage for device fabrication. Concerning the prior technique where the same growth plate form can be used for the fabrication of the device, the bottom-up fabrication techniques are an ideal option instead of the top-down in terms of size, controllability, and reliability. CVD is the reliable method for the NW growth, as it allows the fabrication of epitaxial NWs; also, we can control the dimension of the NWs with the help of these novel techniques which provide specific orientation for the growth of NWs. In comparison with CVD, according to literature [27], InSb NW diameters and aspect ratios can be varied in a large scale in MBE. For the growth of substrate-free NWs, a solution-based synthesis technique is an ideal choice, since this method possesses appropriate variability and controllability of the NW composition with outstanding quality of NWs, is cost effective, and has a high yield of NWs, which makes this techniques very attractive for the production of NWs on a large scale. Figure 3 shows VLS and SLS techniques for the synthesis of NWs with the role of catalyst expressed schematically; more details are given in the following section.

#### Gold as Catalyst

As the prior declaration of Wagner and Ellis [2] in which gold has been used as catalyst for NW growth, it is still no doubt that gold have been used as catalyst materials on a large scale. Of course, gold as catalyst is well renowned and most commonly used as seed particles to enhance the growth of low-dimensional structure, or at least easily available, and directly be used for NW growth; but the question that arises here is that, why Au catalyst is flawless remains unanswered. We will also discuss afterwards that it is productive to take deep scrutiny of the Au–InSb system; as a result, we will determine the standard method that can be used for the metallic catalysts.

First of all, from a purely practical point of view, we will demonstrate few advantages in favor of using gold as catalyst. Availability of Au catalysts makes it more famous as well the due to the extremely low resistance of the gold which makes it attractive for its application during device fabrication especially for electrical contacts. Many research institutes have evaporation systems equipped with gold nanoparticles. Thus, it is easy to deposit thin layer of gold film on sample substrate. Alternatively, one could use colloid nanoparticles with diameters ranging from 1 to 250 nm, which in the case of gold, are easily available (for example see, ref. [70]). High chemical stability and non-hazardous in nature are silent features of gold which make it an ideal candidate for electrode material [13]. Although seemingly trivial, the handling of samples is a most important issue. It is worth noticing to use such catalyst materials that cannot easily oxidize when exposed to ambient, when the regrowth processing of a sample is necessary. Furthermore, the excellent chemical stability of gold nanoparticles [71] decreases many mechanical modifications of the growth chamber, particularly with regard to reducing the background pressure of oxygen. Moreover, for the use of gold as catalyst, the safety requirements are low as gold is comparatively less poisonous [72].

**Fig. 4:**
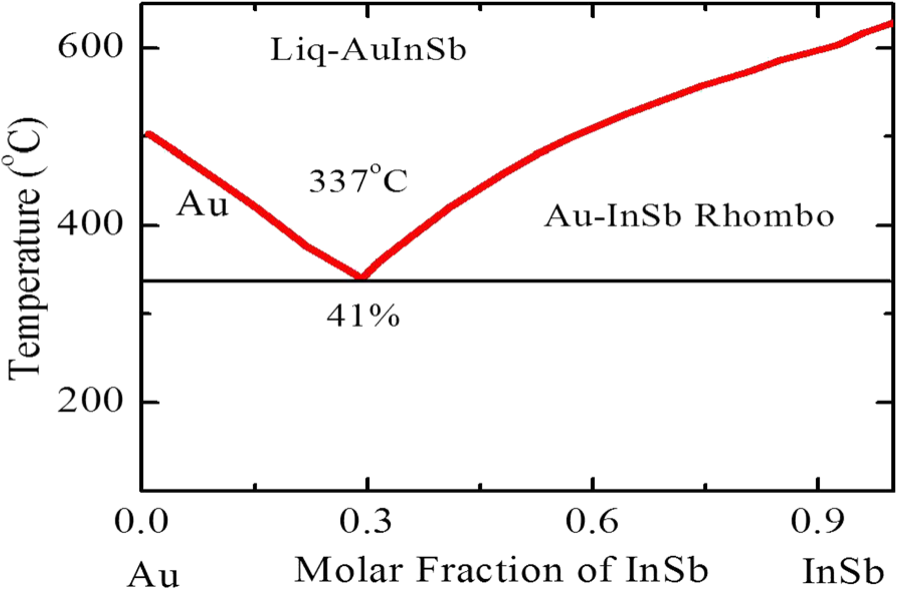
A schematic of the Au–InSb binary phase diagram (PD) experimental DTA points. See ref. [74]

Figure 4 shows the schematic of Au–InSb binary phase diagram (PD) which represent eutectic type behavior of the Au–InSb. This phase diagram represents most prominent characteristics of its eutectic point which is about 41 % InSb at 337 °C as shown in the PD, which demonstrates an extraordinary sudden decrease of the evaporation temperature in contrast to the melting points of pure InSb or pure gold nanoparticles. This means liquefied Au–InSb alloy formed if we anneal a gold-covered InSb nanostructure to a temperature greater than the eutectic point. During this process, due to dewetting instead of homogenous thin film, Au–InSb droplet will be developed on the growth surface. If heating time prolonged, the size of the smaller droplet increased depending upon droplet size, which indicates that with the increase of time the average droplet size enlarged which is known as a phenomenon of Ostwald ripening [73] (for theoretical investigation , see refs [74, 75]). Figure 3 shows that the v-shaped region is actually a liquid phase whose composition is found by the amount of InSb supplied. As gold is abundant and easily available, so we can fabricate Au–InSb alloy droplets on gold-coated substrate. Consequently, the amount of gold for such type of Au–InSb droplets corresponds to a concentration as shown in the PD by InSb-rich liquidus line, generally known as phase boundary which covers the region of the liquidus line that lies on right-hand side. By exposing the substrate to indium and antimony precursors at these elevated temperatures, the precursors, such as TMIn and TMSb, arrive at the surface of the substrate in the vapor phase and solidify due to temperature gradient at the surface of catalysts which germinate NWs. If these continual arrivals of the precursors remain for a long time, these grown nanostructure in the form of bud covert into elongated structures know as NWs. In the phase diagram as shown in Fig. 3, the gold-InSb system, the eutectic point lies on the right side of the v-shaped phase diagram. After the growth of NWs is completed and to get back to equilibrium, catalysts solidify keeping greater concentration of the precursor materials, the stoichiometrical investigation of this phase can be represented on the InSb-rich side of the phase diagram which happens for pure InSb instead of Au–InSb. Growth of InSb NWs can be continued for a long time forcing the system to a non-equilibrium state. Generally speaking, corresponding to metal–InSb PD, growth of InSb NWs depends upon a non-horizontal phase boundary. While the solidification of InSb-rich solid can be enhanced by increasing the precursor pressure which ultimately forced this gold alloy droplet over this phase boundary. To conform that this synthesis is of pure InSb, the phase boundary should be in the neighbor of the pure InSb side. For VLS growth mechanism, this liquidus line corresponds to phase boundary, as indicated in Fig. 3; but this is not necessary. Considering growth of InSb NWs by VSS, which is carried out with a solid catalyst particle, precipitation of InSb takes place at the phase boundary that controls the solubility of metal catalyst nanoparticles with InSb [50, 76]. The most significant characteristic of the Au–InSb binary phase diagram (see Fig. 4) is its comparatively high concentration of InSb 41 % at the eutectic point. Such kind of elevated solubility demonstrates that the energy required to dissolve gold into InSb to form alloy of Au–InSb must be smaller at the eutectic temperature, although InSb possibly dissolve with gold. Therefore, the energetic costs for increasing the concentration of InSb above the equilibrium limit per InSb molecules should also be comparably lowered (also shown by the smaller steepness of the liquidus line). Thus, at the eutectic point, the precursor pressure supposed to have a sufficient boost in the concentration of InSb beyond its equilibrium value that should be lower for metals with a high InSb solubility. Following this supposition, we can deduce that gold is a well-suited catalyst, because one can easily tune the precursor pressure by controlling temperatures. For successful growth of the NWs, solubility of precursors should be strictly monitored; otherwise, it becomes a disadvantage, especially if one aims to grow heterostructure NWs with sharp interface, for example, axial InSb–Ge heterostructures. Lower vapor pressure is another drawback of the gold nanoparticle regarding its catalytic behavior; even at elevated temperatures that is at temperatures of 800 °C, the vapor pressure of gold is less than 8.2 × 10^−8^ Torr. Under ordinary growth conditions of InSb NWs, excess of gold in the chamber is not a critical issue. We also discuss at length why other catalyst materials do not work as efficient as gold, the only reason is high vapor pressure. A major drawback of the gold catalysts is high surface tension of the Au–InSb droplet alloy.

To conclude this section on the phase diagram comprehensively, the major influence of the gold as catalyst is that it is nontoxic, chemically inert, and easily available; low melting point and hence vapor pressure also need to be considered, and solubility of gold in the InSb as well is also considered a lot as it results to a sufficiently high surface tension [56]. Unfortunately, the major drawback of the gold is that it is considered as an impurity in NWs [77, 78], when these NWs are used for further device fabrication. Here, a thorough list that used gold as a catalyst material for the synthesis of InSb NWs has been reported [8, 10–16, 30, 37, 39–41, 44, 52, 53, 55, 56, 62, 68, 79–109].

#### Alternative Catalyst Materials

**Fig. 5:**
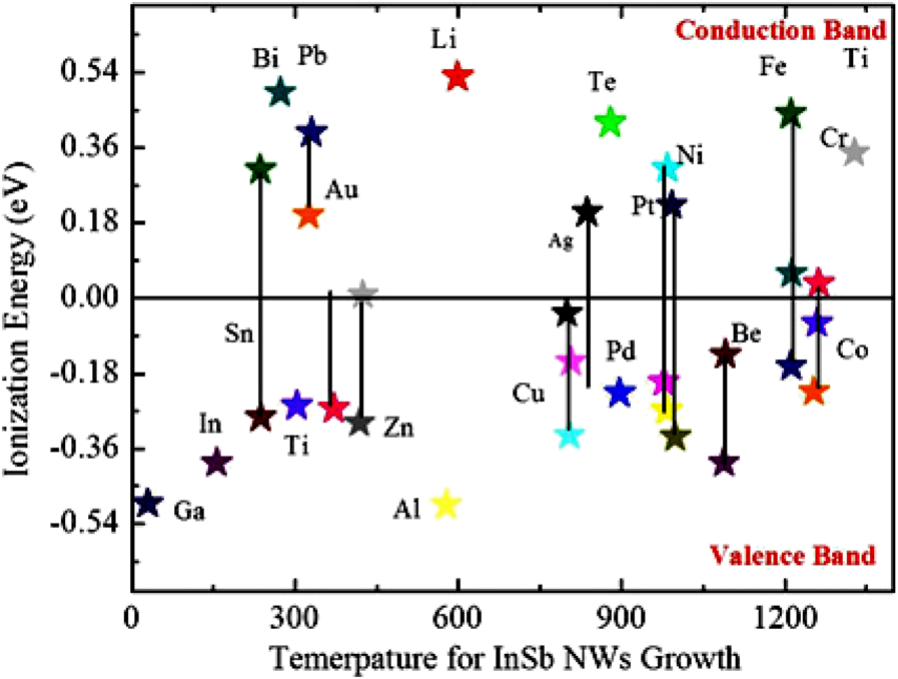
Ionization energies of numerous metal catalysts for InSb NWs (after Capasso [180]) calculated with respect to the middle of the band gap (as band gap of InSb is 0.17 eV) as a function of the growth temperature required for VLS synthesis method. *Connected lines* demonstrated in the phase diagram represent that metal catalyst possess two or more than two levels. Levels *below* the middle are acceptor while *above* are donor

In this decade, a lot of effort has been made to find the non-gold as an alternative catalyst material for NW growth as the gold is not suitable with complementary metal oxide-semiconductor (CMOS) devices [110]. The major issue on why gold is incompatible is actually that it creates defects in InSb to a deep level [18]. This means that gold is an impurity for these devices which significantly affect the lifetime of the charge carries by acting as the recombination center [55]. Maximum recombination rate associated with the impurities like gold within band gap correlated with the energy level of the impurities. To be more concise, it depends on the center of the band gap and energetic difference among the impurity level—the smaller the impurity level in between band gap, the effective it is as a recombination center. Consequently, the insertion of impurities should be avoided whose band gap is close to the middle. With respect to gold, the issue of creating a defect enhances due to its high chemical stability; as a result, one should have to clean prepared NWs from gold which is another big challenge for device fabrication. The impurities whose band gap level is close to the conduction or valence bands of InSb may induce different types of doping depending upon the electronic configurations that is n-doping for Bi, Li, and Te and p-doping for In, Ga, and Al. These materials are attractive for the growth of InSb NWs and, on the other hand, for those catalyst materials whose band gap is in the middle such as Au, Fe, Cr, SiO_2_ Zn, Cu, Pb, or Co. If charge-carrier recombination as well as doping is ignored, then Ni, Ag, Ga, Pt, Sn, Ni, and Pd could be the best choice of catalyst materials, if and only if InSb NWs can be grown by using the abovementioned catalysts. To see whether this is the case, the literature reported on the use of non-Au catalysts can be searched. To summarize all non-gold catalyst data, here is detail list in which other metallic catalysts are used for the growth of InSb NWs reported: SiO_2_ [17–20, 36, 111], Ag [28, 58, 112], Al [113, 114], P [76], In [1, 21–25, 38, 47, 59, 60, 76, 115–118], As [119], InAs [35], Ga [120], Ni [121], Sb [120], Ti [122]. A wide variety of metal catalysts exist which can be differentiated as well as classified in a concise way with respect to the features which generally correspond to binary phase diagram of metal–InSb as shown in Fig. 5.

### Thermodynamics of Nanowire Growth

#### Expansion of the Nanowire

During the initial phase of growth, in the VLS growth mechanism, expansion of the NW base offers a model role for the interaction between seed catalyst and NWs. However, before starting the growth topic, some background knowledge like surface free energy, surface tension, and stress is needed because these terms generally root misperception. These confusions arise because, unlike in liquid crystals, the surface free energy and surface tension or surface stress are not essentially equal. This said statement has already been revealed by Gibbs [123] and later analyzed also by Shuttleworth [124]. The work of creating a new area is called surface free energy; for example by piercing, surface fatigue is related to elastic deformation that is the also known as extension in the surface dimensions [125]. To investigate the properties of this increase in surface area, surface stress tensor can also be used. For isotropic surfaces, this tensor is converted into scalar called surface stress. The author will ignore that distinction by randomly assigning it as the surface tension of the crystalline solids. In addition, the brief overview of line tension was also first revealed by Gibbs in which he discloses [123] that *τ* line tension must be allocated to isolating lines among diverse phases that are completely matched to the theory of surface tension. In focused research work, this describes the dimension of catalyst with indium antimonide interface, where liquid, vapor, and solid phases are associated. At equilibrium conditions, after introducing, the line tension plays a leading role for understanding the term “*t*/*r*” where *r* represents radius of connected line. Limitation of the line expansion is generally assessed in the range 1 × 10^−11^–1 × 10^−9^ Jm^−1^ [126]. So it can be realized that in comparison with surface tension which is commonly 1 Jm^−2^, line tension can be ignored as the radius is too small of a nanometer; even then, it is still not clear that how much line tension plays their role!

This subsection is interesting to sketch the way for our growth and methodology of InSb NWs epitaxially grown on InP substrate with (111)B orientation, namely that InSb NW radius increased in that specified location that is at the heterojunction [10]. In fact, expansion in the body of NWs arises if it is grown by VLS technique. Without recommendation of VLS synthesis technique, one might be curious to consider that this diameter increase has been accredited to an increased particle volume due to the uptake of indium and to the rotation of the NW sidewalls [83, 87]; radial growth is precisely controlled by applying the molarity ratio of V/III, and the droplet volume enlarges due to indium uptake and conclusively to an increase of the NW diameter [10], as phase diagram predicts that AuIn_2_ is a stable phase [127]. An argument, however, speaks against this, put forward by P. Caroff [15] that the weak requirement of the diameter on the axial growth rate specifies surface dissemination from the substrate, which does not affect the InSb NW growth. Gibbs–Thomson mechanism effects on the elemental have potential difference within the seed particle [15]. It is also believed that this diametric increase is caused by the addition of antimony [87]. On the other hand, radiuses of the grown wire scale with the expansion. Therefore, Givargizov deduced that “whisker root expansion was due to over growth which is related to the contact angle symmetry” [128]. It is concluded from the above discussion that contact angle—angle between catalysts and substrates—plays a major role to set the dimensions of the NWs for example for the growth of InSb NWs, the droplet on InP substrates. According to Nebolsin and Shchetinin [129] for stable growth of NWs, contact angle should be greater than 90°. In comparison, as can be realized, for example, in the reported work of S. Vaddiraju [21], it indicates that the increase in the size of the droplet could be due to the change in the wetting behavior and hence in the contact angle between the underlying crystal and molten metal droplet as the growth proceeds [21].

#### Growth Velocity and Gibbs–Thomson Effect

When an atom of the same species is added in the system, an energetic barrier exists known as chemical potential *μ* which is the minimum energy required for an incoming atom to become a part of the existing system. Thermodynamical effects play their role when surface-to-volume ratio is so large that the system under observation is nanoscale, which means that *N* the total number of atoms increases and hence surface area also. A catalyst in general when NWs start to grow is in spherical shape having a volume of 4π/3*R*^3^ − *NΩ* where *R* is the radius and Ω being the volume per atom (assumed to be constant here), the Gibbs-free energy G can be written as G = *μ*_∞_*N* + 4*πR*^2^*δ*, with *δ* being the surface free energy and *μ*_∞_ being the bulk (infinite radius) chemical potential. Using ∂R/∂N = *Ω*/(4*πR*^2^), one can easily find that the chemical potential μ = ∂*G*/∂*N* becomes *μ* = *μ*_∞_ = 2*Ωδ*/*R*. This is the Gibbs–Thomson effect.

The experimental strategy for the growth of cylindrical wire is also the same but one should notice which principles are constant and inconsistent. The Gibbs-free energy of a given morphology of NWs stated as G = *μ*_∞_*N* + 2*πr*^2^ + 2*πrLδ* where *δ* is the surface energy, *r* is the radius, and *L* is the length of NWs. During the fabrication of the NWs, one has to notice only the length while the diameter changes with the ratio of ∂*L*/∂*N* = *Ω*/(*πr*^2^) generally called expansion term while the chemical potential *μ* = (∂*G*/∂*L*)_*r*_ becomes [128]

1 $$ \mu ={\mu}_{\infty }+\frac{2\varOmega \delta }{r} $$

which is a manifestation having resemblance with that for a sphere. From Eq. (1), it can be deduced that the radius of the NWs remain the same but the expansion happens with the factor of 2. If sufficiently long NWs of the order of micrometer in length are considered, even then, factor of 2 still remains and the above equation is validated for that also. For cylindrical NWs in the equation of Gibbs–Thomson, the factor of 2 is still present; this may represent uncertainty which ultimately alters the growth velocity of the NWs, while radius-dependent effects were also observed by L. Lugani et al. [108]

#### Growth Model

The InSb NW synthesized model is established on the material equilibrium relation in the stable state (i.e., under the consideration of time-free supersaturation state in the droplet and at a steady radius *R* = *d*/2 of the indium antimonide NW cylindrical segment) calculated by [130–132]

2 $$ \frac{\uppi {R}^2}{\varOmega_S}\frac{dL}{dt}={\chi}_1J\pi {R}^2\frac{\left(1+ \cos \alpha \right)}{2}-C{J}_{eq}\left(\zeta +1\right)2\pi {R}^2{e}^{R_1/R}+A $$

In the current geometrical pattern, one side of Eq. (2) gives the modification in the quantity of InSb NW pairs in the mentioned tool per unit time, with *L* as subscribed length of the said NWs (indium antimonide stem), Ω_*S*_ symbol depicts the solid volume (elementary) and *t* is the growth time. The partial term on the right-hand side shows the indium flux that openly affects the drop constructing a 90° contact angle to the upper surface of NW, with *J* as the entrance level per unit area, *χ*_1_ as the pyrolysis effectiveness of TMIn at the drop exterior, and *α* as the event angle of the required beam (indium) [131]. The second duration on the left-hand side stances for the desorption from the drop, where *J*_*eq*_ is the steadiness desorption proportion per unit area of the drop (the same as the steadiness desorption proportion from the hard InSb (111) B surface) and ζ is the supersaturation of composite in the bead to the compact condition. The coefficient C in desorption word of Eq. (2) depicts the promising variant of biochemical prospective from its value in the volume alloy for the layer of droplet surface. The investigational feature gives the Gibbs–Thomson (GT) amendment of fluid elemental potential triggered by the curving of the droplet superficial surface, where *R*_1_ = (2*γ*_1*v*_*Ω*_1_)/*K*_*B*_*T* is the distinctive GT radius, *γ*_1*v*_ is the droplet superficial energy, Ω_1_ is the fundamental liquid volume, *T* is the external temperature, and *K*_*B*_ is the Boltzmann constant [131–134]. The ending term of Eq. (2), *A* = *A*^+^ − *A*^−^, assembles donations initiating from the dissemination of sidewall and shallow In adatoms, for which the precise resolution is specified in the previous data [131, 132]. The *A*^+^ word is the dissemination change from the sidewalls to the drop, and *A*^–^ is the opposite flux from the drop. In the current scenario, a direct and simple description of the diffusions can describe the thickness requirements on both extremes. Following the mechanism of [135], here the idyllic case of surface diffusion is under debate which must be noticeably efficient, while nucleation and desorption on the exterior can be ignored. The positive involvement to the diffusion-induced synthesis rate shown, *A*^+^ = *χ*_*S*_*Jπ*[(*R* + *λ*_*S*_)^2^ − *R*^2^]cos *α* + *χ*_*f*_*J*2*RL* sin *α*, contains twin terms. The initial term explains for indium atoms that first impact the diffusion loop of thickness *λ*_*S*_ (called beneath of the effective diffusion length exterior) at the proportion *χ*_*S*_*J* cos *α* and after a while shifted to the drop along the sidewalls, with *χ*_S_ as the pyrolysis effectiveness of TMIn at the superficial surface of substrate. The later factor shows the indium molecules affecting the sidewalls with the specified superficial area considering 2*RL* sin *α* observed by the ray, with the pyrolysis effectiveness *χ*_*f*_. All these molecules contribute in the NW growth if the current diffusion morphology of the indium NWs on the sidewalls is larger than the overall length *L* (which is the summation of the each sidewall lengths of the InSb exteriors (beneath and top). The converse fluctuation in such a direct model must be obtained as ${A}^{+}={J}_{eq}\left(\zeta +1\right){e}^{R_1/R}\left[\pi \left\{{\left(R+{\lambda}_S\right)}^2-{R}^2\right\} \cos \alpha +2RL\kern0.5em  \sin \alpha \right]$, since ${J}_{eq}\left(\zeta +1\right){e}^{R_1/R}$ is the action in the drop improving the superficial diffusion [131, 132, 134]. In the single nuclear manner of NW growth, whereby in each layer only two-dimensional island appears and then fast process scattered to establish a whole single layer slice, the proportion of nucleation-based NW synthesis by virtue of supersaturated composite is described by Dubrovskii et al. [134]

3 $$ \frac{dL}{dt}\alpha \frac{1}{\tau } \exp \left(-\frac{a}{ln\left(\zeta +1\right)-2{\gamma}_{1v}\left({\varOmega}_S-{\varOmega}_1\right)/{K}_BTR}\right) $$

Here *τ* is the specific progression time of level island and *a* is a constant embedded composite related with the superficial surface energy of the island’s horizontal surface, where *R* is the specific parameter (denominator) of the exponent in Eq. (3), which shows the Gibbs–Thomson improvement for the Laplacian pressure in the droplet upon the synthesis of one monolayer slice. It is assumed that [134, 136] the islet scattering is much faster than the waiting time between two corresponding nucleation phenomenons. Let us now assume that *τ* → 0 an ideal example where the nucleation is immediate. To obtain a definite value of $\frac{dL}{dt}$ given by relation (3), we should put the denominator of relation exponent to zero which depicts that,

4 $$ \left(\zeta +1\right){e}^{R_1/R}={e}^{R_{CT}/R} $$

where *R*_*GT*_ = 2*γ*_1*v*_*Ω*_*S*_/*K*_*B*_*T* shows the influence of the Gibbs–Thomson effect on desorption from the drop and the exterior diffusion. Using Eq. (4) in Eq. (2) and in the above expressions for *A*^+^ and *A*^–^, we arrive at

5 $$ \begin{array}{l}\frac{1}{V_{eq}}\frac{dL}{dt}={\chi}_1\left(\phi +1\right)\frac{\left(1+ \cos \alpha \right)}{2}-2C{e}^{R_{CT}/R}+\left(\frac{2{\lambda}_S}{R}+\frac{\lambda_S^2}{R^2}\right)\left[{\chi}_S\left(\phi +1\right) \cos \alpha -{e}^{R_{CT}/R}\right]\\ {}+\frac{2L}{\pi R}\left[{\chi}_f\left(\phi +1\right) \sin \alpha -{e}^{R_{CT}/R}\right]\end{array} $$

Here *V*_*eq*_ = *J*_*eq*_*Ω*_*S*_ is the steady state desorption rate and *ϕ* = *J*/*J*_*eq*_ − 1 is the saturation in the current suspension segment w.r.t. the solid state. As described in [130], our direct and efficient equation comprises the 1/*R* diffusion span due to the exterior adatoms, while the dissemination of superficial surface adatoms is defined by the 1/*R* and 1/*R*^2^ relations ascending differently with the radius. Also, the dissemination relationship is altered to demonstrate the significant correct way of diffusion flow rates provisional on the action in the drop. The use of estimation of immediate nucleation permits us to abolish the unknown *ζ*, thus eluding a difficult technique of balancing the entering physical flux of indium particles by their nucleation-mediated droplet [137].

#### Surface Energies and Crystal Structure of Nanowire

**Fig. 6:**
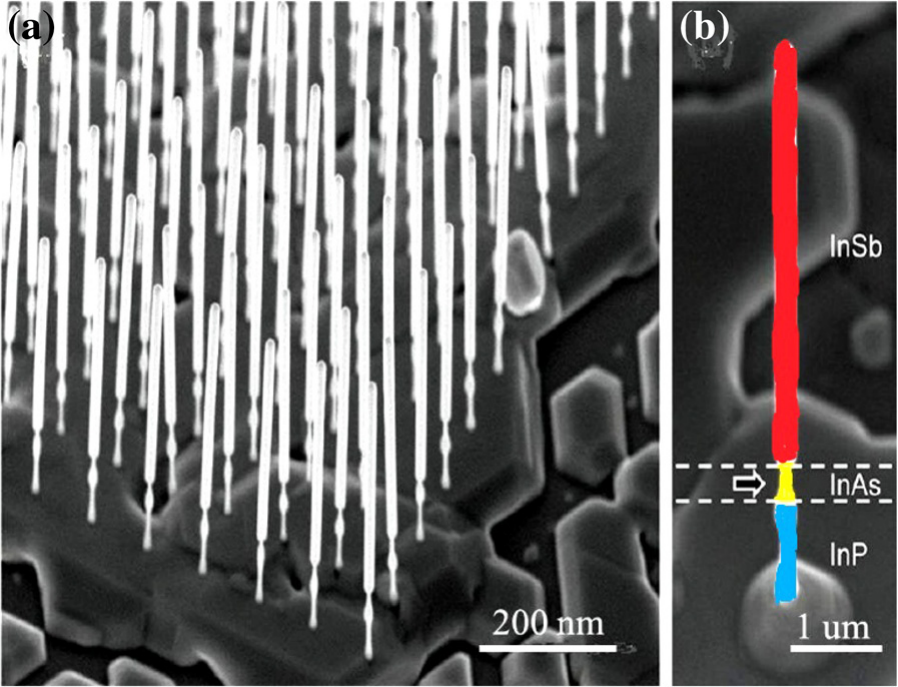
The 30° tilted SEM images of an InSb NW array. **a** Low-magnification image of a 25 × 25 NW array with a yield of over 95 % grown using optimized parameters for 50-nm droplets: pitch = 500 nm and V/III = 26.4. *Scale bar* corresponds to 1 μm. **b** High magnification image of a single NW. Color indicates the stacking of materials: InP (*blue*), InAs (*yellow*), and InSb (*red*). The *arrow* shows the zone in which the InAs stem evaporates. *Scale bar* corresponds to 200 nm. See ref. [10]

As reported in [138–140], the synthesis of (111) oriented NWs having wurtzite (WZ) or zinc blend (ZB) configurations of III–V semiconductor materials was due to dangling bonds on side walls which cause the decrease in surface energy. At the triple phase line where nucleation occurred [141], there is a significant difference between surface energies of WZ and ZB islands which is greater for ZB which causes nucleation barrier; although for WZ phase in bulk, it has higher cohesive energy [134, 141, 142]. As a result, to start the nucleation process, this difference of energies should be overcome. To understand the crystal structure and faceting of NWs, we get information from the surface energies of (110) and (211) ZB planes and their respective $\left\langle 1\overline{1}00\right\rangle$ and $\left\langle 2\overline{1}\overline{1}0\right\rangle$ WZ counterparts; after dissection of ZB and WZ III–V crystals, the analysis of dangling bond can be carried out [143, 144]. The ZB–WZ planes corresponding to $(211)-\left(1\overline{1}00\right)$ and $\left\langle 110\right\rangle -\left\langle 2\overline{1}\overline{1}0\right\rangle$ WZ–ZB differ by 30° of rotation axis, and these NWs have regular hexagonal symmetries from whom more detailed analysis of nearest atoms in the lattice was also carried out [144] (Fig. 6). As in [138, 140, 143], to conclude this part, the surface energy produce by dissection of planes can be measured by dangling bond density per unit area *a* [2] where it is known as lattice constant of the dissected crystal. These planes are represented as

6 $$ \gamma ={\varphi}_{\mathrm{hkl}}={n}_1{\varphi}_1+{n}_2{\varphi}_2 $$

where *φ*_1_ and *φ*_2_ are the interaction energies between neighbor atoms and *n*_1_ and *n*_2_ are _the_ total number of neighbor atoms along dissected ZB plane while (hkl) are the Miller’s indices of that plane by ignoring the surface reconstruction. To calculate the surface energy of the (111) and (100) ZB planes, the above procedure is adopted for whom interaction energies are known for InAs and InSb crystals [145, 146]. Accordingly, the surface energies of the NW facets such as $(211),\kern0.5em (110),\kern0.5em \left(1\overline{1}00\right),\kern0.5em \mathrm{and}\kern0.5em \left(2\overline{1}\overline{1}0\right)$ can also be calculated whose detail can be found in [144]. Using the crystal structure and self-consistent theory of NW growth reported in [143], nucleation-mediated growth rate includes effective chemical potentials of catalysts as well as surface energies with growth rate after balancing source materials whose details are explained in Eq. (5). Here, one can easily calculate critical radius at which the growth rate is equal for both ZB and WZ structures after doing some mathematical work as reported in [144], separately. These calculations are performed for six $(110)\kern0.5em \mathrm{and}\kern0.5em \left(1\overline{1}00\right)$ facets and WZ/ZB InAs NWs that have lowest surface energies. Critical radius is a key parameter which decides which phase dominates either WZ or ZB; above *R*_C_, ZB phase prevails while below *R*_C_ WZ phase dominates. For these calculations, at the island-catalyst interface, the surface energy *γ*_*S*1_ of liquid–solid is cited in [134], where *γ*_*WZ*_ = *γ*_(1 − 10)_ and *γ*_*ZB*_ = *γ*_(110)_ are the surface energies of ZB and WZ NW sidewalls respectively and *T* corresponds to growth temperature. The difference in the cohesive energies of ZB and WZ phases of NWs represented by *ψ* are reported in [147]. Crystalline structure of NWs grown is another promising feature related with III–V semiconductor materials [24, 148, 149] that leads to stacking faults, and planner defects which may reduce the efficiency of devices have been investigated [150]. Therefore, many groups successfully grew NWs with pure phase such as zinc blend or wurtzite. As phase changes, band gap is modified, which results in potential applications [151–153] due to different optical and electronic properties. In fact, some novel structures like zinc blend or wurtzite or zinc blend twinning superlattice have been investigated [82, 154–156]. However, antimonide NWs with the ZB structure are dominating from the previous reports, and this keeps true even for ternary antimonide NWs with only a small Sb concentration [157]. Although Mandl et al. [24] and Pozuelo et al. [76] have reported WZ structure of antimonide NWs. there is still neither theoretical prediction nor experimental data about wurtzite structured antimonide NWs.

### Electrical and Optical Properties of InSb NWs

Physical properties of InSb NWs need attention after their successful growth. In this section, optical and electrical properties and hence device fabrication from InSb NWs have been thoroughly discussed. We also review applications of InSb NWs in optoelectronic industry for the fabrication of nanodevices such as infrared detectors and field-effect transistors to explore the basic physics in depth.

#### Electrical Properties

A number of the growth mechanisms have been adopted and successful for the growth of InSb NWs; after this successful growth, electrical and optical properties have also been studied a lot [21, 61]. Figure 13 shows the electrical characteristic measured from these grown NWs. It is a very interesting finding that InSb NWs exhibit p-type conduction with mobility of 57 cm^2^ V^−1^ s^−1^ while the thin film of InSb shows n-type behavior with sufficiently high mobility of 1200 cm^2^ V^−1^ s^−1^ grown by electrodeposited method and annealed at 420 °C for few hours [13]. By using the CBE method to grow InSb NWs, these grown NWs with an evaluated resistivity exhibit n-type conduction of 0.3 Ωcm at ambient. Thermally dependent resistivity was attributed to carrier generation across the band gap whose activation energy is equal to the half of the band gap [58]. Based on the synthesis techniques for InSb NWs, the performance of field-effect transistors (FET) based on InSb NW has been evaluated. Device fabrication from these NWs was carried out, and it is revealed from the transport characterization measurement that mobility is sufficiently high than InAs [11]. On the other hand, excellent transport properties of InSb NWs at low temperature (4 K) are observed, with electron concentration of 1 × 10^17^ cm^−3^ at field-effect mobility of 35,000 cm^2^ V^−1^ s^−1^ [10]. Coherence length of the order of 260 nm was also observed. It is a fact that the FETs based on InSb NWs are superior in performance than those fabricated from InAs NWs which are due to two major reasons, pinning of surface Fermi level and Schottky barrier. It is noticed that in InAs-based FETs, Fermi level pins into conduction band which results in negligible small resistance [158]. On the other hand, InSb NWs typically show pinning of Fermi level close to the valence band as a result Schottky barriers develop at the contacts [159].

**Fig. 7:**
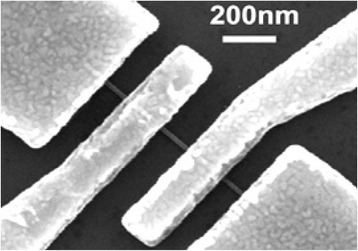
A scanning electron microscope image of a representative back-gated InSb NWs. See ref. [17]

**Fig. 8:**
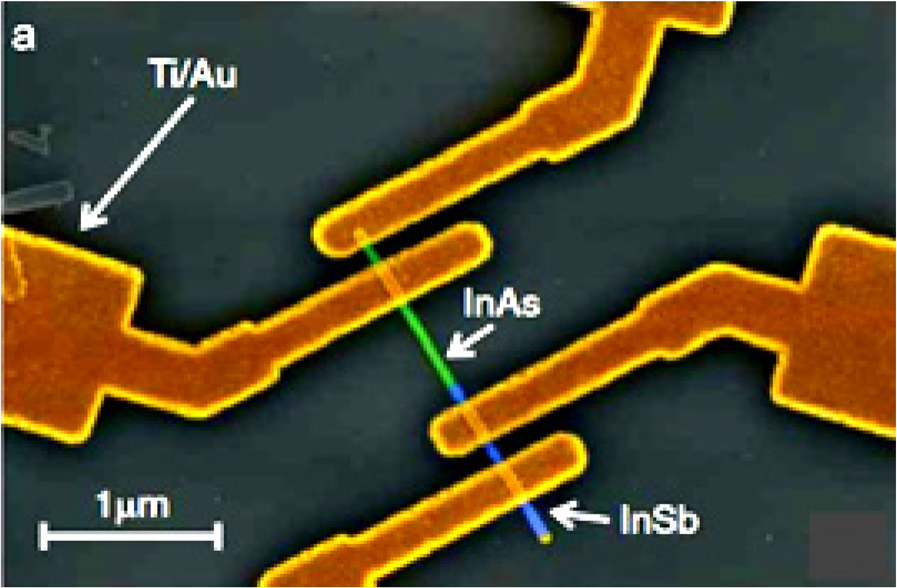
Colored SEM image of InAs/InSb heterojunction. See ref. [177]

Huijun Yao et al. [17] fabricated InSb NW-based back-gate field-effect transistor. By comparing the performance of InSb NW-based FETs with various diameters, they observe that bulk transport dominates as shown in Fig. 7. Owing to the interface states with large electron concentration, only a small modulation in the NW resistance with back-gate voltage is observed. This forecasts that in the NWs, a metal-like degenerate electron gas exists, which depends on the temperature for NW conductance. The emergence of these fluctuations of conductance at low temperatures confirms the existence of electron interference [17]. Transport properties of InAsSb NWs have been investigated as function of composition as shown in Fig. 8. It is observed that field-effect mobility and transconductance increase significantly when the concentration of antimony increased in comparison to InAs NW-based FETs. It is deduced that this improved performance of the device was due to shift of crystal structure from defect-rich WZ phase to defect-free ZB phase for which mobility of 3500 cm^2^ V^−1^ s^−1^ is measured at room temperature [157].

#### Band Gaps and Band Alignments

InSb NWs have distinct physical properties revealed by simple optical reflectance measurements and analogy with the identical results from a bulk InSb wafer; it is obvious that antireflection coating can be fabricated from such a NW arrays and could be useful for photon management studies and nanostructured energy harvesting [90].

Photoluminescence measurements were carried out to investigate the optical properties of antimonide NWs. Band gap of antimonide NWs corresponds to bulk of semiconductor materials while ZB phase dominate in their crystalline structure. Infrared optical absorption spectroscopy revealed the band gap of InSb NW arrays. These NWs with a radius of 40 nm revealed a detection edge of about 170 meV whereas higher edge of 200 meV is observed for those NWs having diameter 60 nm [62]. These decreases in diameters exhibit a blue shift that was attributed to confinement effects. Existence of a lot number of heterojunction in the NW geometry were due to the relaxed constraint of lattice matching, and material integrations such as InAs/InSb, InAs/GaInAs/GaSb, InAs/GaSb, InAs/InP/InSb, and NWs that have already been developed. Bulk synthesis of InSb/InAs heterojunction NWs and related material combinations is difficult. During fabrication of heterojunctions in NWs, band alignment can be changed due to change in phase for example in InAs NWs both ZB and WZ phases are equally possible. Another unique type of heterostructures is created during growth of ZB InAs_0.9_Sb_0.1_ on GaSb substrate [160]. During growth of InAsSb NWs, antimony has been incorporated from substrate which may affect electrical properties. Theoretical approximations, for InAsSb NWs, the band structure proposed show a valence band off-set of about 495 meV with a band gap of 270 meV [161], while the experimental observations in this off-set of energy is quite different, which is impossible to measure due to transport mechanism at low source-drain bias.

#### Device Structures

##### Field-Effect Transistors

**Fig. 9:**
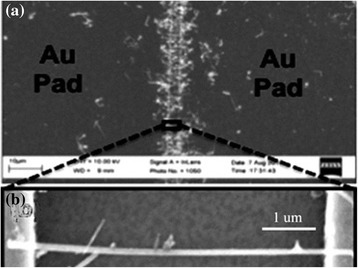
**a** SEM image of the aligned InSb NWs bridging the source-drain electrodes after ac electrophoresis alignment. **b** Enlarged SEM image of an individual suspended InSb NWs between electrodes. See ref. [16]

FETs based on the InSb NWs attract a lot of attention due to its high electron velocity and hence overall mobility of the device. Indeed, at reduced drive power, these FETs based on InSb NWs exhibit comparatively superior performance [162]. It is obvious to use InSb NWs for the fabrication of FETs because of promising device feature such as high current saturation and good transconductance [11, 13, 163]. A narrow band gap is a drawback of InSb NWs which decreases the amount of bias applied to FETs based on InSb NWs at the drain/gate interface, where the electric field is strong which causes bipolarity in device features and ultimately enhances the off-state current. For sensor applications, back-gated FETs based on InSb NWs have successfully demonstrated the detection of poisonous gasses [16]. When this device is exposed to poisonous gas such as NO_2_, their resistance increases which can be measured and their sensitivity under detection limit of 1 ppm is 1.05 % per ppm for NO_2_. This experimentally revealed decrease in resistivity was due to transfer of charge from NO_2_ molecules to the InSb NW surface. The schematic scanning electron microscopy image of fabricated FET for the detection of NO_2_ gas is shown in Fig. 9.

**Fig. 10:**
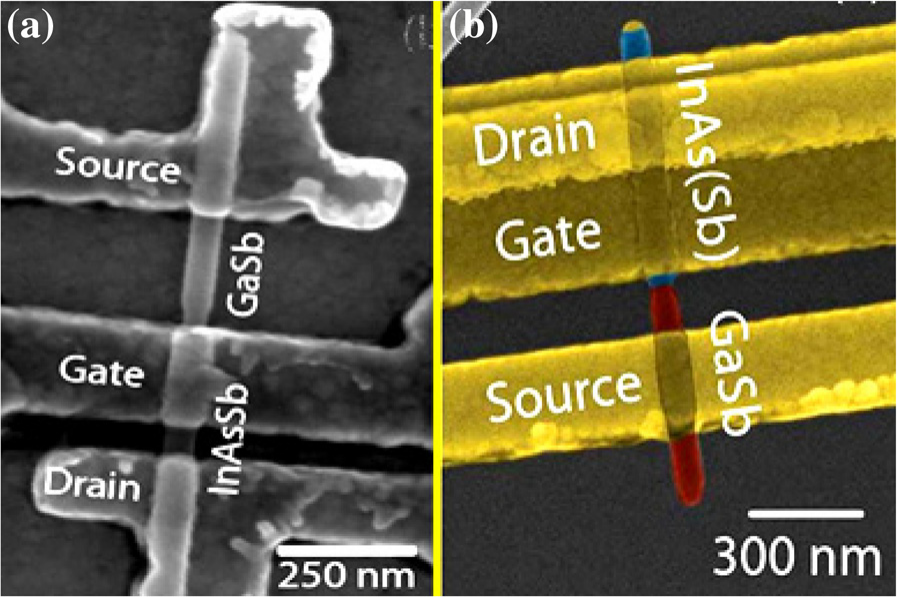
**a** SEM image of an InAsSb/GaSb TFET device with electrode configuration for reverse biased operation. See ref. [169]. **b** Colorized scanning electron microscopy image of a TFET. Note that the apparent NW diameter is here increased by the HfO_2_ film and that the gate overlap varies between devices. See ref. [170]

InSb NWs are also attractive for the synthesis of tunneling field-effect transistors (TFETs) [164]. In a common TFET design, potential on the gate electrode can be used to modulate tunneling current at p-n interface. As a consequence, TFETs can be used for energy filtration in which only charges with small energies are transmitted; gate voltage less than 60 mV/dec is the maximum limit for traditional FETs which has advantageous property being low dimensional [165, 166], and gate electrode used for the modulation of potential in the channel. Barrier tunneling is interesting feature for type II-heterojunction fabrication to enhance the current in TFETs. Current level may also been enhanced because of the materials such as InSb with low effective mass [167, 168]. It has recently been observed that a broken type-II alignment is more preferable than staggered alignment. Even though the current level is greater than broken gap alignment, still it is believed that off-set current decreases in fabricated device. So far, InAsSb/GaSb NW heterostructures show high current density when tunnel diode is fabricated from it [101, 160]. Recently, GaSb/InAsSb heterojunction NWs have been used for the fabrication TFETs which exhibit high current as shown in the Fig. 10a, b [169, 170]. Due to a non-optimized gate process, these devices, however, still deteriorate in their off-characteristics. It is observed that numerous combinations of In(Ga)As and AlGaSb NWs have been under investigation to avoid the overlapping of valance band into conduction band.

##### Quantum Devices

**Fig. 11:**
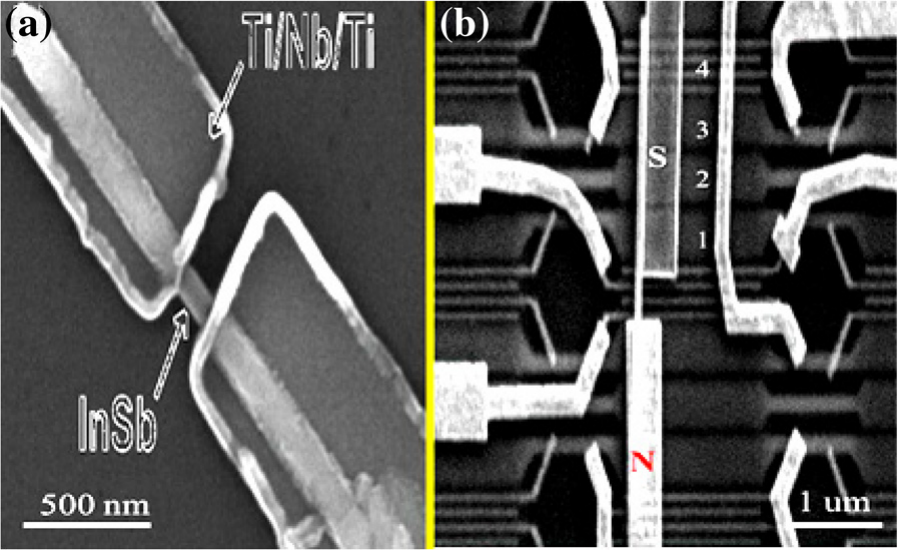
**a** SEM image of the fabricated Nb−InSb NW−Nb junction device. In this device, the diameter of the InSb NW is about 65 nm, the separation between the two Nb-based contacts is about 110 nm, and the lengths of the InSb NW sections covered by the two Nb-based contacts are about 740 and 680 nm, respectively. See ref. [172]. **b** Scanning electron microscope image of the device with normal (*N*) and superconducting (*S*) contacts. The *S* contact only covers the right part of the NWs. The underlying gates, numbered 1 to 4, are covered with a dielectric. [Note that gate 1 connects two gates, and gate 4 connects four narrow gates. See ref. [171]

Magnificent mobility of 35,000 cm^2^ V^−1^ s^−1^ has been measured at low temperature from InSb NWs showing as an excellent candidate for ideal transport characteristics [10]. Furthermore, large g-factor >50 for InSb NWs is an attractive material for magnetic measurements. It is found that energy levels also affect the magnitude of g-factor [49]. As the value of g-factor changes under the influence of magnetic field, so it can be utilized to study the energy level crossing having same spin orientations, as well as it can be measured by the suppression of the tunneling current feature of FETs. InSb NWs were also used to fabricate the semiconductor-superconductor device at atomic scale to reveal Majorana Fermion by using sophisticated instrumentation [171, 172]. Two independent groups used such kind of devices for the observation of Majorana Fermions originating from junctions [171, 172]. Scanning electron microscopic images of the devices are shown in Fig. 11a, b. These outstanding results can only be obtained from NW geometry of InSb because it has large spin-orbit interactions. Obviously, novel quantum phenomena can be observed with one-dimensional InSb as it is a promising material due to its band structure.

##### Thermoelectrics

Thermoelectric applications of InSb NWs have been investigated due to its low thermal conductivity and the high electron mobility. The major logic for this decrease in thermal conductivity is the reduction in phonon density which may decrease the thermal conductivity of NWs. In this material system, higher than three thermoelectric figures have been predicted [173]. The thermoelectric power can be generated due to difference of temperature known as Seebeck effect, and the Luttinger Liquid Theory has been used to study the conductivity of the these low-dimensional material [174, 175] which proposes power law dependency of conductance on heat. Furthermore, both the Seebeck coefficient and conductance shift strongly on the temperature and changes when temperature changed. Luttinger liquid theory explains transport properties of the InSb NWs which predict that this extraordinary mobility was due to these transitions [1]. Moreover, thicker NWs (radius of 20 nm) exhibit a lower Seebeck coefficient related to thinner (with the radius of 10 nm) NWs. Major causes of the thermopower in these InSb NWs are electron-electron interaction as well as back scattering of impurities.

##### Detectors

A chemiresistive FET for the detection of NO_2_ gas grown by chemical vapor deposition (CVD)-based InSb NWs as it has narrow band gap of 0.18 eV at 300 K has been reported, and scanning electron microscopic image of FET is shown in Fig. 9a, b. When this device operated at room temperature, the fabricated sensor can detect NO_2_ down to 1 ppm, which is five times smaller than Occupational Safety and Health Administration (OSHA) permissible limit of 5 ppm. The high sensitivity of the sensors corresponding to the working principle of fabricated device in which resistance of device depends on variation in concentrations of NO_2_ exposures [16].

**Fig. 12:**
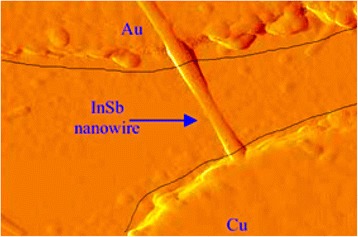
Atomic force microscopy (AFM) image of the InSb NW IR sensor with unbalanced metal electrodes. A single InSb NW sorts relations to Au and Cu electrodes on a quartz substrate. See ref. [181]

Two independent research groups reported the fabrication of M-IR photodetectors using individual InSb NWs [93, 176] based on the metal-semiconductor-metal assembly shown in Fig. 12. It is observed that the requirement of the photocurrent on the strength concentration follow power law, which predict the reality of defect states that are reliable with an n-type conductivity phenomenon in the InSb NWs. Furthermore, these photodetectors reveal good photoconductive performance, reproducibility, good stability, and superior responsivity of 8.4 × 10^4^ AW^−1^, and quantum efficiency (1.96 × 106 %). These distinctive features are ascribed to the superior crsytallinity and high surface-to-volume ratio of InSb NWs [93].

The thin band gap for indium antimonide alloy characteristics from the said materials is very attractive for optoelectronic device applications. The strategic purpose for introducing NWs is the possibility for physical incorporation and the choice of different NW combination systems as the limitations of lattice similarity are minimized. The optical features for heterostructured constituents and hence the device fabrication were not demonstrated at the moment. Even many advancements in NW fabrication techniques and diverse applications in many well-developed field were done up to a certain extent. Phase segregation and hence their ordering may play a vital role in the said NWs and this feature requires many development. Integration of lattice-mismatched can be achieved for NWs with typically small diameters, but also the light coupling efficiency decreases at some specific optimized wavelengths for focused fabricated detectors (>5 μm). Svensson et al. [79] investigated recently on light absorption efficacy of InAsSb NW heterostructures while ternary structures grown on the stem of the InAs NWs having a radius of 25–50 nm which causes elastic strain relaxation, after getting an ideal growth condition, the authors are able to grow sufficiently long NWs with the length of 700 nm.

**Fig. 13:**
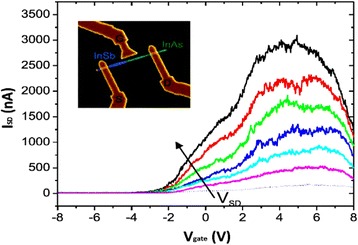
*I*_*sd*_ − *V*_gate_ at various source-drain voltages, within range of 10-60 mV. In the *inset*, scanning electron microscopic (SEM) image of fabricated device active region is depicted. The coupled arms of the log-periodic antenna recognized via e-beam lithography are correspondingly, associated to source and gate electrodes, while the current is examined with the aid of drain (*D*) one. See ref. [122]

As a consequence, light absorption efficiency improved at wavelengths greater than 5 μm. Another important group of devices (diodes) are the detectors for THz, and millimeter wave detections that operate in reverse bias with suitable NW manufacturing can exhibit their solid responsivity even at zero bias. InSb/InAs heterostructured NWs fabricated by MOVPE and CBE [163, 177] demonstrating a solid modification effect caused by band twisting at the hetero-interface (Fig. 13). The detector responsivity, described as the relation between the *V*_sd_ with respect to second- to first-order derivative of current, was reported by Pitanti et al. [177] who attain the value of about 5. More precisely, to obtain the responsivity near zero bias [178] as well as to cut down the component of thermionic current, authors introduce a thin film barrier (2–5 nm) of InP thin film barriers at the heterojunction. As a result, fabricated devices are charming for low value noise detector employments. It has been experimentally demonstrated for the detection of THz radiation in InAs/InSb NW heterostructure-based FETs (shown in inset of Fig. 13) while this device responsivity is comparable to the one found in InAs-based FETs [122].

## Conclusions

To sum up, we have noticed that there are numerous techniques to grow InSb NWs using metallic catalysts or self-seeding. Among these catalysts, gold is a promising material due to its inertness with a few drawbacks, as gold is introduced inside NWs that appear as defects which is not desirable. Alloys of InAsSb, GaAsSb, GaInSb, and InPSb NW heterostructures were revealed with their lattice-mismatched having also been recognized. It is expected that advance individual crystalline NWs and doped form of the said NWs will be fabricated soon, which will open a new horizon in the nanotechnology and more detailed studies of the quantum phenomenon. For example, no research studied has been made on fabrication of AlGaSb, AlSb, and AlInSb NWs that is antimonide NWs containing aluminum as a ternary material. Such materials show promising characteristics due to variability in its band gap by introduction of ternary alloy which can be up to 1.62 eV. The introduction of aluminum for heterostructure growth may cause oxidation due to chemical reaction, and as a result, may affect the catalyst activity as result growth rate decreases. Despite these facts, antimonide NWs containing aluminum will be have the scope needed to be explored for future generation optoelectronic devices. Depending upon these applications of antimonide NWs, few major aspects of these materials are promising, and these are large g-factor and investigation of spin-orbit interaction to reveal quantum phenomenon. Particularly, investigation based on NW devices is a unique way to understand low-dimensional systems. Other characteristic features of antimonide NW-based devices are to use as detectors and emitters in the infrared regime of electromagnetic spectrum offered by NW technology. For the realization of its complete potential, further heterostructured NW improvement is, however, essential/prerequisite. Dopants are used to enhance device performance and to control conductivity. In addition, surface passivation methods to decrease surface-related opportunistic recombination and conduction as well as advance contact schemes are required. To decrease the formation of intrinsic imperfections in the material, growth parameters required further optimization; as a result, optical and electrical characteristics improved as well. Two-dimensional InSb also grown by MBE have been reported. These thin films are free standing on the InAs NWs using buffer-layer engineering. The length and width of the grown InSb can be controlled by tuning the Sb/In beam evaporation ratio with ZB single crystals. It is expected that plentiful comprehensive materials reported that united with empirical device applications will continue to push the advancement in InSb NW performance and quality; the heterostructured NWs will provide the opportunity to young scientists to contribute their research in this era which should be helpful to improve the awareness and understanding of these inimitable NWs.
